# Comparison of Cyberbullying before and after the COVID-19 Pandemic in Korea

**DOI:** 10.3390/ijerph181910085

**Published:** 2021-09-25

**Authors:** So Young Shin, Yeon-Jun Choi

**Affiliations:** 1 Department of Police Administration, Joongbu University, Geumsan 32713, Korea; agilescv@joongbu.ac.kr; 2 Department of Aviation Security Protection, Kwangju Women’s University, Gwangju 62396, Korea

**Keywords:** cyberbullying, perpetrating experience, victimization experience, school bullying, COVID-19

## Abstract

Because of the implementation of social distancing measures following the onset of the COVID-19 pandemic, face-to-face interaction has plummeted, which has resulted in the prolific use of social networking services (SNS) and increased activity in the cyberspace environment. This is especially true for teenagers and young adults with the shift to online classes in the education sector, which has increased the chances of being exposed to cyberbullying. This study attempts to determine a strategy for counteracting cyberbullying in the post-COVID-19 era by identifying the factors that have contributed toward greater aggression by adolescents in South Korea in 2020 when the spread of COVID-19 was at its height. To achieve this, we employed the Cyberbullying Circumstance Analysis dataset from the Korea Communications Commission for the time frame of between 2019 and 2020, with 4779 and 4958 participants, respectively. The causes and effects that led to cyberbullying were investigated using binary logistic regression analysis. By reviewing the research data targeted towards Korean adolescents, our analysis found that the average age of those who engage in cyberbullying decreased in 2020 compared to 2019. In addition, cyberbullying awareness at school and the school’s capability of controlling it influenced the number of cyberbullies from a statistical grounding, which could be found only in the 2020 dataset. An approach to establishing policies in schools to contain preemptive measures and increase cyberbullying awareness has been proposed to prevent such mishaps in the post-COVID-19 era.

## 1. Introduction

Every age group in Korean society is becoming information-focused with the availability of diverse media content and the ubiquity of smartphones in daily life. In an effort to stem the widespread transmission of COVID-19, the Korean government implemented social distancing measures, including restrictions on social gatherings and events and the expansion of work-from-home policies. This resulted in an inverse relationship between real life and cyberspace interactions, with a decrease in the former and an increase in the latter. Another ramification of a physically distant society is the elevated importance of cyber platforms and digital media, which has spurred their utilization by journalists as well as in the social and economic sectors. As a result, various negative consequences have appeared and are increasing from the acceleration of the information era, including cyber-ostracization, verbal abuse, personal information leaks, and defamation [1,2,3,4]. Cyberbullying—intentionally excluding a particular person or continually harassing a person or group in the cyber environment—has been gaining attention as a social problem that is related to the stay-at-home period of the COVID-19 pandemic, as it has been rapidly increasing. A key characteristic of cyberbullying is that people do not recognize it as an act of violent aggression nor do they realize that exposure to it is not limited to school environments, but that rather, students are covertly harassed or abused twenty-four hours a day, making it a challenge to detect and repress. Hence, it has become a serious problem practiced by adolescents at school [5]. In other words, acts of abuse at school continue in the form of cyberbullying after school with victims having little ability to end the vicious cycle. It is no surprise that the victims face everyday problems, such as refusing to go to school or poor academic performance but are also subject to psychological difficulties that include depression and suicidal urges [6]. In some respects, this problem is much more malicious than it may appear because adolescents are not equipped to handle the situation, unlike adults, and they are in the stage of life when it is easier to give in to their emotions, which could lead to the spawn of another abuser or making a decision that proves to be fatal [7]. However, social distancing measures have increased remote or online education, which in turn has propagated further reliance on online platforms. This phenomenon can be observed in South Korea, where there is a higher chance that adolescents will be exposed to cyberbullying compared to the pre-COVID-19 period. Considering the unique characteristics of cyberbullying, we have come to the conclusion that cyberbullying among adolescents has been exacerbated when compared with the time before the COVID-19 pandemic, since there is now a more active use of cyber platforms and SNS because of social distancing measures. With a specific focus on the year 2020 (when COVID-19 was at its height), we have analyzed the factors that contributed to making South Korean students more predisposed to become cyberbullies. We analyzed the differences in the factors affecting the experiences of cyberbullying perpetrators between 2019 and 2020. Our research suggests the direction for the policies responding to cyberbullying in the post-COVID-19 era.

## 2. Issues in Cyberbullying

### 2.1. The Definition of Cyberbullying and Its Unique Characteristics

The definition of cyberbullying is in dispute among scholars; however, they do seem to agree that it is an act of abuse via a digital device or an information platform. In essence, cyberbullying refers to abusive behavior that occurs in cyberspace, and it is not limited to defamation and humiliation, but also extends to hate speech and stalking [8]. Having researched the subject of cyberbullying for an extensive period, Hinduja and Patchin [9] define it as an “act of harassment of friends through the medium of chatrooms or social networking websites.” Additionally, the characteristic features of cyberbullying are not accidental and ephemeral but deliberate and persistent [10]. A key difference with offline violence lies in the fact that the perpetrator does not have to be in proximate contact with the victim, and thus, the violence level becomes much greater [11,12] and, also results in adolescents not perceiving the severity of cyberbullying. They have difficulty distinguishing humor and playfulness from abusive behavior, privacy infringement, or constant taunting [7]. This phenomenon is remarkable especially for the younger age group, and they tend to solve troubles in friendships online. [13]. In addition, those engaged in cyberbullying experience it as both perpetrator and victim. For example, in a study by Beran et al. [14] that involved 1001 Canadian adolescents from the age of 10 to 17, 14.0% (140 pupils) had fallen victim to cyberbullying more than once in the previous month and 8.0% (80 pupils) had been the perpetrators. Of the 140 victims, 25.7% (36 pupils) had also been abusers, and numerous studies describe the overlapping roles of victim and abuser in cyberbullying [15,16,17].

### 2.2. The Effects of COVID-19 on Cyberbullying

The current situation with cyberbullying in South Korea shows a decrease in in-person violence at school but an increase in cyberbullying, and this trend applies not only to South Korea but throughout the world. Schneider et al. [18] conducted a study involving 16,000 high school students to determine how school violence and cyberbullying progressed during the time frame between 2006 and 2012. They reported that in 2006, offline school violence was 1.7 times as frequent as cyberbullying (26% and 15%, respectively), but in 2012 the two forms of abuse were equally prevalent (23% and 21%, respectively): offline school violence had decreased by 3% while cyberbullying had increased by 6%. According to Schneider et al. [18], who surveyed 20,406 students in grades nine to twelve, 15.8% of them had experienced cyberbullying, with 25.9% of them having experienced offline bullying in school. Furthermore, 59.7% of the cyberbullying victims were also victims of offline school abuse, while 36.3% of these victims were cyberbullying offenders. From this, it can be deduced that cyberbullying is increasing amongst adolescents. Many studies [19,20,21,22] explain that this phenomenon has a high association with the usage of smartphones; they particularly focus on the increasing rate of ownership of smartphones by adolescents as well as the fact that interpersonal relationships are connected to various activities online. Face-to-face social activities were largely unavailable because of social distancing restrictions during the COVID-19 pandemic. The repercussion was the more intense formation of interpersonal relationships in the cyber environment as well as on SNS. The development and expansion of accessibility to the cyber platform and digital media have resulted in the proliferation of cyberbullying, meaning that the conditions have been met for it to enter a new phase.

## 3. Research Methods

### 3.1. Data Collection

In order to determine the proportion of Korean students who have experienced cyberbullying, we utilized the State of Cyber Abuse Survey data from the Korea Communications Commission for the period from 2019 to 2020. The survey is based on schools’ statistical data from the South Korean government and uses stratified systematic sampling by school grade distinction, geographical location, and grade. We focused on the correlation between internet usage behavior and cyber abuse of elementary, middle, and high school students. The method and scope of the research are as follows: the survey area included the 17 cities/provinces across South Korea where, in the case of elementary students, those in the higher grades of 4 to 6 being capable of understanding and answering the survey were subject to the research. Middle and high school students were selected through the stratified systematic sampling, where the schools were selected and then the classes were chosen arbitrarily. In 2019, there were 4779 pupils where the data was collected from 1 October 2019 until 23 November of the same year, while in 2020, the number was 4958 between the time period of 6 October 2020 and 13 November 2020.

The method of data collection was to select a number of schools from a summarized list and to send the survey—as well as a guide explaining how to complete it—by mail in 2019. However, the survey was moved online in 2020 because of the COVID-19 pandemic, but the method remained the same as in 2019: specific schools from the summarized list were contacted, and the teacher in charge provided the link to the survey to students. This type of stratified sampling is a method that creates homogeneous sub-groups according to specific key characteristics from the parent population and extracts samples. For this to be possible, adequate information about the parent population must be known, which requires additional cost and time. The advantage, however, is that the homogeneity of such groups reduces sampling error and facilitates the identification of the key characteristics of the relevant variables. The general attributes of the subjects in the State of Cyber Abuse Survey are listed in Table 1.

### 3.2. Measurement

The dependent variable in this research paper represents the cyberbullying experience, and we have defined it as any action that causes distress to another person in the cyber environment (such as internet, cellular phone) via language, video among other aspects. For each cyberbullying action, we have labeled the measured variable as either “experienced” or “non-experienced” (Cronbach’s a >0.8). In addition, the dependent variable has been converted to a dummy value of either a 0 (non-experienced) or 1 (experienced) so that it can be used for the binary logit analysis.

As for the different forms of cyberbullying, the following explains the trait of each one. Cyber verbal abuse constitutes slander, aggressive language, statements of personal attack, etc. Cyber defamation is the up-loading of text, regardless of its validity, that inflicts damage on the reputation of other people/institutions on the internet or Social Networking Services so that anyone (non-specific mass of people) is able to view it. Cyberstalking means sending e-mails or messages, visiting blogs or Social Networking Services, leaving a comment, anything that marks a trace, thereby constantly rousing emotions of fear and anxiety even though the victim does not wish such acts. Cyber sexual abuse represents behavior that are sexual imitations or are statements of sexual belittlement, gender discrimination, or any content that makes the other person feel sexual distress and that is posted on the internet through a medium such as a smartphone, or the distribution of lewd pictures or videos. Cyber ostracization is the mentioning or posting of one’s personal life, secrets, etc., on the internet or Social Networking Services. It also includes the dissemination of personal information (name, residential address, name of school that one goes to, etc.) as well as ostracizing one in internet chat rooms or instant messages. Cyber extortion is the act of stealing cyber money (including virtual currencies in games) or data in smartphones, etc. Cyber coercion is the practice of forcing one to say/do acts that one has no intention of doing through the medium of the internet or cellular phone or giving orders to do the abuser’s bidding.

The independent variables were selected based on the precedent established by an earlier study [23] and include the following factors: awareness of cyberbullying, frequency of exposure to harmful content, number of friends perpetrating cyberbullying, reliability of friendships, parent–child interaction, school involvement in limiting cyberbullying, and observation of whether or not one had witnessed cyberbullying. The awareness of cyberbullying measures the student’s awareness that sanctions may be imposed on cyberbullies, as well as the potential for legal sanctions, thereby serving as a recognition of the seriousness of cyberbullying. The exposure to harmful content measures the frequency of contact with violence, sensationalism, slander, illicit behavior, false advertising, online gambling, and other forms of online peer pressure. The reliability of friendship and parent–child interaction measures the levels of trust and interactivity between friends and family members. School involvement quantifies the degree to which schools and teachers participate in programs and regulations associated with cyberbullying prevention as well as their interest in it. Lastly, the observation variable tests the validity of having witnessed an event of cyberbullying during the span of one year (Table 2).

### 3.3. Analytic Strategies

To clarify the factors that impact the perpetrator’s experience of cyberbullying among South Korean students, we coded and cleaned the collected data before commencing with the analysis. In this study, we conducted a frequency analysis to confirm the attributes of the sample. In addition, we have produced a Cronbach’s α value through the reliability test to assess the reliability of the variables. The correlation analysis was also executed so that the correlation among the variables could be verified, as well as the binary logit analysis for the establishment of a cause-and-effect relationship. The survey results were analyzed using the Statistical Package for the Social Sciences (SPSS) WIN 18.0 program (IBM, Armonk, NY, USA).

## 4. Results

### 4.1. The Experience Rate of Cyberbullying of South Korean Students before and after the Onset of COVID-19

The change of experience of cyberbullying was described in Table 3. After comparing the experience rate of cyberbullying between 2019 and 2020, we found that in 2019, the rate was 26.9%, while in 2020, the rate (perpetrator or victim) was 22.8%, which is a 4.1% drop from the previous year. The perpetration rate of cyberbullying in 2020 was 9.5%, which is a sharp decline of 8.5% compared to the previous year, but there was a slight increase of 0.7% in the victimization rate.

In addition, as shown in Table 4, analyzing the data according to the students’ gender and school grade distinction resulted in a drop in perpetration and observation rate by 8.5% and 6.9%, respectively, along with a decline of 3.7% in the “Both perpetration and victimization” field. Looking at the change in the cyberbullying experience rate in 2020 in comparison to 2019 from the school grade distinction viewpoint, the middle school and high school perpetration experience rates had decreased, while those rates were on a similar level as elementary schools. The victimization experience rate of elementary school students had increased by 6.9%.

### 4.2. The Relationship between the Cyberbullying Perpetrator Experience and the Main Variables

To confirm the correlation between the cyberbullying perpetrator experience and the main variables, we conducted a bivariate correlation analysis and the results were exhibited in Table 5. Here, we found a meaningful statistical correlation between each of the independent variables and the cyberbullying perpetrator experience in the 2020 dataset. However, in the 2019 dataset, we were not able to identify any correlation between the awareness of the seriousness of cyberbullying and the school’s involvement in curtailing cyberbullying.

### 4.3. Comparison of Factors That Affect the Cyberbullying Perpetrator Experience before and after COVID-19

To compare the factors that affected the cyberbullying perpetrator experience on South Korean students before and after the onset of COVID-19, we inserted the main variables. The analytical output of the assumed logit model is presented in Table 6.

Several conclusions may be drawn from this analysis. First, examining the data from 2019, it is clear that the factors of gender, frequency of exposure to harmful content, number of friends perpetrating cyberbullying, friendship reliability, parent–child interaction, and experience of having observed cyberbullying influence the cyberbullying perpetrator experience on a statistically significant level. In contrast, examining the 2020 data, it is clear that the factors of gender, educational stage (school grade distinction), awareness of cyberbullying issues, exposure to harmful content, number of friends perpetrating cyberbullying, parent–child interaction, school involvement, and cyberbullying observation affected the cyberbullying perpetrator experience on a statistically significant level. In other words, during 2019, being of the male gender, having a higher frequency of contact with harmful content, having more friends perpetrating cyberbullying, exhibiting lower friendship reliability and lower parent–child interaction, and observing cyberbullying bring about a cyberbullying perpetrator experience. In 2020, after COVID-19 was prevalent, the following factors were significant to the cyberbullying perpetrator experience: male gender, a lower educational stage (elementary school), lower awareness of cyberbullying issues, a higher frequency of exposure to harmful content, more friends perpetrating cyberbullying, lower parent–child interaction, lower school involvement, and cyberbullying observation.

Gender, frequency of exposure to harmful content, number of friends perpetrating cyberbullying, parent–child interaction, and cyberbullying observation experience all had an impact on the cyberbullying perpetrator experience, regardless of the presence of COVID-19. Friendship reliability had an effect only in the 2019 dataset, while educational stage, awareness of cyberbullying issues, and school involvement had an effect on the cyberbullying perpetrator experience in the 2020 dataset only. It is apparent that the case of females having a cyberbullying perpetrator experience was 49% lower than that of males and decreased even further to 61% in 2020. In the case of parent–child interaction, the cyberbullying perpetrator experience when low interaction was present was 1.522 times greater in 2019 and 1.526 times higher in 2020, respectively, than that from high interaction. In terms of the differences relating to the educational stage after the COVID-19 pandemic, we could find no effect of it in Model 1, but in Model 2, the educational stage had increased by one level. This resulted in a 47% reduction in cyberbullying perpetrator experiences.

## 5. Discussion

The following conclusions may be drawn by comparing and analyzing the cyberbullying perpetrator experiences of South Korean students based on the existing data prior to the spread of COVID-19 in 2019 and after the spread in 2020.

First, we compared and analyzed the various factors that had an effect on cyberbullying perpetrator experiences before and after COVID-19 with regard to South Korean adolescents. Gender, frequency of exposure to harmful content, number of friends perpetrating cyberbullying, parent–child interaction, and cyberbullying observation were the factors that had a statistically significant effect. In numerous earlier studies [16,24,25,26,27,28], the abovementioned factors had a meaningful effect on cyberbullying, and they continued to do so during the COVID-19 pandemic.

Second, the cyberbullying experience rate of South Korean students decreased between 2019 and 2020, but the victim experience rate increased conversely. At first glance, the cyberbullying perpetrator experience rate could be regarded as a decrease, but previous studies, [15,16,17] consistently suggest that cyberbullying perpetration and victimization may overlap; thus, the relation between perpetration and victimization experiences should be considered. In other words, although either the cyberbullying perpetrator or victimization experience decreases, it is necessary to consider diverse external factors, unless both of the experiences decrease simultaneously to a meaningful extent. Considering the 2020 dataset with regard to the educational stage, it was found that the perpetrator experience had decreased in the case of middle school and high school students, but was fairly constant for elementary school students. However, for the victimization experience, there was an increase compared to the previous year for elementary school students. Consequently, the average age of experiencing cyberbullying among South Korean adolescents is on a downward trend. It is expected that this phenomenon is closely related to smartphone ownership. Most middle and high school students own smartphones, which has brought about an increase in cyberbullying prevention programs, whereas the relatively low possibility of owning a smartphone by elementary school students has led to a lack of education in preventing cyberbullying. However, with the start of the COVID-19 pandemic and the onset of an online education environment nationwide, the ratio of elementary school students possessing a smartphone or laptop and the time spent using such devices has increased abruptly, which has accelerated the aforementioned phenomenon.

Third, when examining the factors that had a distinct effect on cyberbullying perpetrator experiences before and after the start of COVID-19 (2019 and 2020), friendship reliability did have an impact in the first year, but we could not find any meaningful effect in the second year. A possible explanation for this is that in 2020, most South Korean schools had already adapted to online lessons, which would have limited the amount of involvement and contact among friends. It has already been demonstrated in earlier research [29,30] that friendship reliability is a major factor for explaining juvenile delinquency, hence, is mentioned in this paper.

In contrast, the educational stage, awareness of cyberbullying issues, and school involvement had a statistically significant impact only in the 2020 data, or after the spread of COVID-19. Of these, the educational stage factor (as mentioned previously) is a result of contributing circumstances that tend to lower the average age of experiencing cyberbullying after the onset of COVID-19. Unlike pre-COVID-19 times, the expanding influence of the awareness of cyberbullying issues factor can be traced to people’s heightened interest in matters in the cyberspace, which increased due to remote education. Moreover, this study attempts to prove that a school’s involvement in controlling cyberbullying has become more important in the post-COVID era when the remote learning environment has been utilized. Tangen and Campbell [31] point out a real-world problem is that school teachers tend to focus on superficial offline school violence and they do not pay attention to hidden violence such as cyberbullying, even to the point of intentionally avoiding getting involved in it. However, in the world of online classes, teachers focus more on cyber violence than offline school violence, and it seems that this combined effort resulted in the school involvement having a stronger impact on cyberbullying perpetrator experiences.

In conclusion, there has been a considerable shift in the factors that have an effect on the cyberbullying perpetrator experiences of South Korean students before and after the spread of COVID-19. South Korea is laying the foundation for a remote education system and is planning to expand its non-contact learning platform in its mid- and long-term plan to adapt to post-COVID times after the pandemic subsides. Therefore, it is necessary for the South Korean government to alter its plans based on the results of this research. For example, considering that the average age of experiencing cyberbullying is decreasing after the onset of COVID-19, the government should develop educational programs targeted to elementary school students and should make the programs mandatory for all applicable schools. Additionally, considering that awareness of cyberbullying issues and school involvement have become more critical factors after the initial spread of COVID-19, schools and teachers should abandon the common assumption that cyberbullying is an extension of offline school violence and realize that more proactive and preemptive action is required.

With the significance of such research, we would like to propose future research beyond the limitations of this one. First, the data was collected based on the school statistical data from the national Korean Educational Development Institute that were aimed at the elementary, middle, and high school students where it was carried out according to school grade distinction, geographical location, and grade through the stratified systematic sampling, making it more exemplary than non-probabilistic sampling. However, it still falls short of not being a longitudinal study in addition to the fact that the subjects of the research in 2019 and 2020 were different. It is recommended that forthcoming studies necessitate research sampling to overcome the error of generalization. Second, the research explores the topic from a wide variety of angles without distinguishing those that have influenced the harm from cyberbullying, but future research should look into the distinction between the harm done that is specific and that is not. This is so the mechanism of harm from violence can be better understood as well as being a more objective source of material. Last, there is a need for extra research on the relationship of the arbitrator of school violence with the inclusion of longitudinal datasets to extend the horizon of the research.

## Figures and Tables

**Table 1:** Socio-demographic characteristics of the participants.

Year	2019			2020		
	Range	*n*	%	Range	*n*	%
Gender	Male (=1)	2589	54.2	Male	2568	51.8
	Female (=2)	2190	45.8	Female	2390	48.2
Education Level	Elementary school (=1)	1577	33.0	Elementary school (=1)	1738	35.1
	Middle school (=2)	1560	32.6	Middle school (=2)	1645	33.2
	High school (=3)	1642	34.4	High school (=3)	1575	31.8
Total		4779	100		4958	100

**Table 2:** Descriptive statistics of the variables.

Year	2019			2020		
	Range	*n*	%	Range	*n*	%
Awareness of cyberbullying	High	4619	96.7	High	2759	55.6
	Low	160	3.3	Low	2199	44.4
Exposure to harmful content	High	622	13.0	High	510	10.3
	Middle	837	17.5	Middle	1354	27.3
	Low	3320	69.5	Low	3094	62.4
Number of friends perpetrating cyberbullying	None	4343	90.9	None	4728	95.4
	1~3	339	7.1	1~3	198	4.0
	Over 4	97	2.0	Over 4	32	0.6
Friendship reliability	High	4368	91.4	High	4536	91.5
	Low	411	8.6	Low	422	8.5
Parent–child interaction	High	4289	89.7	High	4518	91.1
	Low	490	10.3	Low	440	8.9
School involvement	High	1148	24.0	High	1217	24.5
	Middle	2336	48.9	Middle	2527	51.0
	Low	1295	27.1	Low	1214	24.5
Observation	Experienced	767	16.0	Experienced	453	9.1
	Non- experienced	4012	84.0	Non-experienced	4505	90.9
Perpetration	Non- experienced	3919	82.0	Non-experienced	4489	90.5
	Experienced	860	18.0	Experienced	469	9.5
Total		4779	100		4958	100

**Table 3:** Comparison in experience rate of cyberbullying.

Year	2019	2020
*n*	4779	4958
Perpetration or victimization	26.9%	22.8%
Perpetration	18.0%	9.5%
Victimization	19.0%	19.7%
Both perpetration and victimization	10.1%	6.4%

**Table 4:** Change in cyberbullying experience rate in 2020 compared to 2019.

	Total	Male	Female	Elementary School	Middle School	High School
*n*	4958	2568	2390	1738	1645	1575
Perpetration	9.5 (−8.5)	13.1 (−8.4)	5.5 (−8.3)	12.4 (−0.9)	9.3 (−13.8)	6.3 (−11.3)
Victimization	19.7 (0.7)	22.0 (3.9)	17.2 (−2.8)	25.8 (6.9)	18.1 (−4.8)	14.7 (−0.7)
Both perpetration and victimization	6.4 (−3.7)	8.3 (−3.5)	4.3 (−3.6)	9.7 (1.8)	5.8 (−7.8)	3.4 (−5.4)
Observation	9.1 (−6.9)	9.1 (5.7)	9.2 (−8.4)	12.3 (−3.9)	7.6 (−9.3)	7.3 (−7.9)

**Table 5:** Bivariate relationships between variables.

	Model 1 (2019)								Model 2 (2020)							
	1	2	3	4	5	6	7	8	1	2	3	4	5	6	7	8
1	1								1							
2	−0.018	1							0.147 **	1						
3	−0.235 **	0.081 **	1						−0.149 **	−0.049 **	1					
4	0.214 **	0.016	−0.123 **	1					0.163 **	0.066 **	−0.107 **	1				
5	0.068 **	0.109 **	0.039 **	0.108 **	1				0.059 **	0.094 **	0.021	0.011	1			
6	0.079 **	0.125 **	−0.054 **	0.043 **	0.211 **	1			0.088 **	0.127 **	−0.018	0.039 **	0.289 **	1		
7	0.001	0.075 **	−0.075 **	−0.001	0.023	0.067 **	1		0.032 **	0.103 **	−0.059 **	−0.029 *	0.056 **	0.089 **	1	
8	−0.223 **	0.012	0.191 **	−0.262 **	−0.043 **	−0.057 **	0.039 **	1	−0.192 **	−0.052 **	0.120 **	−0.246 **	−0.041 **	−0.096 **	0.011	1

Note. 1. Perpetration experience, 2. Awareness of cyberbullying issues, 3. Exposure to harmful content, 4. Number of friends perpetrating cyberbullying, 5. Friendship reliability, 6. Parent–child interaction, 7. School involvement, 8. Cyberbullying observation (* *p* ≤ 0.05; ** *p* ≤ 0.01).

**Table 6:** Factors affecting experiences in cyberbullying perpetration before and after COVID-19 pandemic.

		Model 1 (2019)			Model 2 (2020)		
		B	S.E.	Odds Ratio	B	S.E.	Odds Ratio
Gender		−0.673 ***	0.086	0.510	−0.954 ***	0.115	0.385
Educational stage		−0.095	0.056	0.909	−0.646 ***	0.075	0.524
Awareness of cyberbullying issues		−0.371	0.251	0.690	0.765 ***	0.110	2.149
Exposure to harmful content		−0.697 ***	0.056	0.498	−0.841 ***	0.077	0.431
Number of friends perpetrating cyberbullying		0.731 ***	0.091	2.078	0.730 ***	0.145	2.074
Friendship reliability		0.354 **	0.136	1.424	0.133	0.165	1.142
Parent–child interaction		0.420 **	0.123	1.522	0.423 **	0.155	1.526
School involvement		−0.043	0.059	0.958	0.174 *	0.078	1.190
Cyberbullying observation		−0.915 ***	0.097	0.401	−1.048 ***	0.135	0.351
Constant		1.738 ***	0.442	5.686	1.264 **	0.543	3.541
−2Log likelihood		3964.006			2611.630		
Cox and Snell’s R^2^		0.107			0.095		
Nagelkerke R^2^		0.175			0.203		
χ^2^		540.906 ***			492.486 ***		
Accuracy		82.9%			90.6%		

Note. * *p* ≤ 0.05; ** *p* ≤ 0.01; *** *p* ≤ 0.001.

## Data Availability

The data presented in this study are available on request from the corresponding author.
